# Assessment of malaria diagnostic methods and treatments at a Ghanaian health facility

**DOI:** 10.11604/pamj.2021.39.251.28996

**Published:** 2021-08-19

**Authors:** James Kojo Prah, Samuel Amoah, Andrew Nicholas Yartey, Adelaide Ampofo-Asiama, Elvis Ofori Ameyaw

**Affiliations:** 1 University of Cape Coast Hospital, Cape Coast, Ghana,; 2 School of Pharmacy and Pharmaceutical Sciences, University of Cape Coast, Cape Coast, Ghana

**Keywords:** Diagnosis, malaria, microscopy, routine laboratory staff, Ghana

## Abstract

**Introduction:**

it has been more than a decade since the World Health Organization (WHO) recommended parasitological confirmation of malaria before treatment begins. Light microscopy and rapid diagnostic tests are currently being used for diagnosing malaria in routine clinical care settings. Many clinicians have however raised questions about the competencies of laboratory staff who perform these tests and the performance of these diagnostic methods. This study aimed at assessing the performance of microscopy and two rapid diagnostic test kits in the hands of routine laboratory staff compared to expert microscopy as well as assess the performance of clinical diagnosis.

**Methods:**

this was a cross sectional study involving 799 participants of all ages who visited the out patient department of the University of Cape Coast Hospital with symptoms suggestive of malaria.

**Results:**

when the different methods were compared to expert microscopy, the rapid diagnostic test kits had the highest sensitivities, Wondfo 94.83% (95% CI: 85.62-98.20) and CareStart 91.38 (95% CI: 81.02-97.14). Microscopy by laboratory staff had a sensitivity of 68.79 (95% CI: 55.46-80.46) whilst clinical diagnosis had the lowest sensitivity of 17.24 (95% CI: 8.59-29.43). Cohen´s kappa coefficient was used to measure the level of agreement of the methods with expert microscopy. Microscopy by laboratory staff, CareStart and Wondfo showed substantial measures of agreement (k = 0.737, 0.683, and 0.691 respectively).

**Conclusion:**

these findings suggest that clinical diagnosis is highly unreliable whilst rapid diagnostic tests and microscopy performed by routine laboratory staff could be trusted by clinicians as reliable diagnostic methods.

## Introduction

Malaria is hyper-endemic in Ghana and it present to the country, a serious health problem. According to the 2019 Ghana malaria indicator survey [1] malaria positivity among children aged 6 months to 59 months using microscopy was 14% in the country. There are two main ways of diagnosing malaria. These are clinical and laboratory diagnosis. Clinical diagnosis is when clinicians diagnose malaria based mainly on patients´ signs and symptoms without a test. Laboratory diagnosis on the other hand, involves identifying malaria parasites and or antigens/products in the blood of patients. Malaria rapid diagnostic tests (RDT) and microscopy are routinely used to diagnose malaria in the clinical setting. In early 2010 however, WHO issued revised treatment guidelines that called for a shift from presumptive to test-based approach [2]. This revision to the guidelines effectively brought to an end the practices of several decades. In 2010, Ghana adopted WHO´s test-based management of malaria [3]. It was therefore expected that all prescribers confirm all suspected cases of malaria before commencing treatment. There has over the years been a mis-match between policy and practice. This situation caused the country failure to achieve its set target of reducing by 75% malaria morbidity and mortality and to test all suspected cases of malaria by 2020 [4]. In the past, sporadic and inadequate availability of rapid diagnostic tests and microscopy [5-7], reliance on strong clinical suspicion of malaria [5,8], mistrust in the performance of microscopy and rapid diagnostic tests in diagnosing malaria [5,9] and a lack of alternative diagnosis [9] have led clinicians to presumptively treat malaria in febrile patients without parasitological evidence.

Since many clinicians continue to trust their clinical suspicion, and have concerns about the reliability of the various rapid diagnostic test kits and the expertise of health facility workers who perform microscopy, there is a need to determine the accuracy of clinical diagnosis compared with laboratory diagnosis. Performance of RDTs compared to microscopy has been studied under different settings globally with variable findings. However, most of these studies have assessed the performance of these diagnostic methods in the hands of expert microscopists and other specially trained study staff. Only few studies [10] have provided estimates of RDT and microscopy performance among hospital laboratory staff. There is therefore the need to assess the performance of two different RDTs and microscopy in the hands of health facility workers involved in routine clinical management of malaria in Cape Coast, a highly endemic region of Ghana. The main aim of this paper was to assess the performance of malaria diagnostic methods at the University of Cape Coast Hospital. This was specifically done by determining the prevalence of malaria among out-patients according to clinical diagnosis, microscopy and the use of two different malaria rapid diagnostic test kits. The sensitivity and specificity of microscopy and each of the malaria rapid diagnostic test kits as performed by laboratory staff were determined using expert microscopy as gold standard.

## Methods

**Study design**: this was an analytical cross-sectional hospital-based study conducted from March 2020 through May 2020.

**Population**: the study involved patients who visited the out patient department of the University of Cape Coast (UCC) Hospital and were either diagnosed clinically of malaria or sent to the laboratory on suspicion of malaria. The UCC Hospital is located on the University of Cape Coast campus, northern part of Cape Coast, Ghana.

**Sampling:** participants were recruited based on the following inclusion and exclusion criteria.

**Inclusion criteria:** all patients irrespective of age and sex, suspected of malaria infection and asked to report to the laboratory with a malaria test (microscopy and/or RDT) request form. Also, all patients irrespective of age and sex who present to the pharmacy with a prescription for an anti-malaria drug without prior malaria testing were recruited.

**Exclusion criteria:** excluded were all patients taking anti-malarial drugs at the time of study or have taken anti-malarial drugs within 2 weeks prior to reporting to the hospital.

**Sample size calculation:** sample size was calculated using 95% confidence, an estimated malaria prevalence of 18% [1] and precision level of 5%. The formula: n = Z^2^ P(1-P)/d^2^ where n = sample size, Z = Z statistic for a level of confidence, P = expected prevalence or proportion and d = precision was used for the sample size calculations. The resulting minimum sample size required was 226. However, in order to increase the statistical power of the study a final total sample size of 799 was used.

**Sampling technique and recruitment:** about 25 patients report every day for a malaria test at the UCC hospital laboratory. Systematic random sampling technique was used to recruit 8 consented patients into the study every day using a sampling interval of 3. In order to reduce the possible coercive influence of clinicians on patients´ acceptance to be part of the study, the recruitment was done by 2 (two) trained research assistants who approached potential participants for their consent whilst they were waiting for their turn at the laboratory. Also, all patients who report to the pharmacy with a malaria prescription without prior malaria test were recruited by the attending pharmacist/pharmacy technician using census sampling. Microscopy and rapid diagnostic tests using two different RDT kits were performed for each participant by hospital laboratory staff as well as experts.

**Data collection procedures:** a questionnaire was used to collect data on demographic characteristics of participants through an interview. A data extraction form was used to document test results and adherence to test results by clinicians.

**Blood sampling:** approximately 3mL of blood samples was taken by trained study staff into an ethylene diamine tetra acetate (EDTA) tubes using the venous sampling technique.

**Rapid diagnostic tests:** about 0.5mL of the blood was used to perform the malaria RDT tests following the manufacturer´s protocols. The RDT kits used were *P. falciparum* specific CareStart malaria HRP2 Test Kit and pan-specific Wondfo malaria HRP2/pLDH test kit.

**Microscopy:** about 1mL of the same blood was used to prepare the thick and thin films which were stained with Giemsa. After laboratory staffs have performed the tests, two experts using the same samples prepared and read blood film slides independently. All laboratory work was conducted at the laboratory of the University of Cape Coast Hospital. Thick blood films were used to estimate the level of parasitaemia by counting the number of parasites against 200 white blood cells with the assumption that each subject had 8,000 white blood cells/µL of blood. A minimum of 200 fields was examined before declaring slides negative for malaria parasite. Blood smears with discordant expert microscopist results (differences in species diagnosis, discrepancies in the parasite density of >50% or disagreement on presence/absence of parasites) was re-examined by a third independent microscopist, and parasite densities calculated by averaging the two closest counts.

**Haemoglobin determination:** about 0.5mL of blood was used to determine the haemoglobin level of participants.

**Ethical consideration:** ethical clearance was obtained from the institutional review board of University of Cape Coast (UCCIRB). Permission was also obtained from the management of the University of Cape Coast Hospital before sampling began. The main areas of concern in ethical involvement with participants included the issues of privacy, anonymity, confidentiality and safety of patients. These issues were addressed by training all those involved in the study to ensure confidentiality. Also, no names, or any form of personal identification was used. A coding system was used to identify participants. Samples collected from participants were handled solely by trained laboratory staffs. Subjects diagnosed with malaria were treated according to the Ghana Ministry of Health guidelines.

**Data analysis:** data collected was entered into Microsoft Excel and imported into SPSS version 23.0 for analysis. All patients prescribed anti-malaria drugs without a laboratory test was categorized clinically diagnosed. Using expert microscopy as gold standard, the sensitivities and specificities of clinical diagnosis, microscopy and rapid diagnostic tests (two different kits) performed by hospital laboratory were determined. Chi-square was used to analyze associations between patient characteristics such as age and sex and malaria test results. Cohen´s kappa coefficient was used to measure the level of agreement between the various methods (CareStart and microscopy; CareStart and Wondfo; Wondfo and microscopy) methods. P-values less than 0.05 was considered significant.

## Results

A total of 799 participants aged 3 months to 86 years were involved in the study. Most participants (55.9%) were females. The average age of participants was 23.9 ± 17.7 years. In general, males (25.02±16.6 years) were older than females (22.62±19.00 years) with no significant difference in age, t(797)=1.898, p=0.058, between them. Children under-five years involved in the study were 147 (18.4%). Table 1 shows the age and sex distribution among participants. The mean haemoglobin level among participants was 12.24±1.77g/dl with a minimum of 4.2g/dl and a maximum of 18.2g/dl. The mean haemoglobin among males (12.82±1.98g/dl) was higher than that of females (11.79±1.43g/dl) with no significant difference, t(797)=-8.527, p=0.058.

**Table 1:** age and sex distribution of participants

Age group (years)	Female (%)	Male (%)	Total
0-10	92	120	212
11-20	96	57	153
21-30	120	73	193
31-40	65	45	110
41-50	33	22	55
51-60	25	18	43
61-70	13	8	21
>70	3	9	11
TOTAL	447	352	799

**Prevalence of malaria:** using expert microscopy as a gold standard a prevalence rate of 7.3% (58/799) was found among the study population. Among these, 11(19.0%) were children under 5 years old. There was no significant association between expect microscopy positivity and age group (X^2^=0.13, p=0.91) as well as sex (X^2^=0.15, p=0.90). there was a higher preponderance of malaria parasitaemia among females (55.2%) compared with males (44.2%). This difference was however not statistically significant (X^2^=0.015, p=0.902). Malaria prevalence varied among the population using the different diagnostic tools. The least prevalence was obtained using laboratory staff performed microscopy as shown in Table 2. The distribution of malaria parasite species identified in the study are as follows: *P. falciparum* only 54(93.1%), *P. falciparum* and *P. malariae* mixed infections, 2(3.4%) and *P. malariae* only 2(3.4%). Thus *P. falciparum* accounted for majority (96.6%) of all cases in this study. The geometric mean of asexual parasitaemia was 14663.34 parasites/µl (range: 123-102220 parasites/µl). When age was stratified into <5 years and >5 years, there was no significant association (X^2^=2.14, p=0.14) between parasite density and age.

**Table 2:** malaria prevalence using different diagnostic methods

Method	Frequency (f)	Prevalence (f/799)
Laboratory staff performed microscopy	48	6.00
Expert microscopy	58	7.26
CareStart RDT	110	13.76
Clinical diagnosis	110	13.76
Wondfo RDT	115	14.39
RDT: rapid diagnostic tests		

**Predictive indices of the various diagnostic tests:** using expert microscopy as the gold standard, the CareStart and Wondfo RDT kits recorded the highest sensitivities of 91.38% and 94.83% respectively. The highest specificity of 98.92% was recorded by microscopy performed by laboratory staff. Clinical diagnosis recorded the poorest sensitivity of 17.24%. Table 3 shows the sensitivities and specificities of clinical diagnosis, CareStart RDT, Wondfo RDT and microscopy performed by laboratory staff at 95% confidence intervals (CI). There was a significant association between expert microscopy and microscopy performed by laboratory staff (p<0.001, by Fisher´s Exact) which was not modified by whether a participant was under 5 years or not (Breslow-Day, 0.800) and by the sex of the participant (Breslow-Day, 0.943). When a logistic regression was performed, both age (aOR: 0.99 (0.983-1.016), p-0.925) and sex (aOR: 0.711 (0.896-1.27), p-0.25) did not significantly predict slide positivity by laboratory staff.

**Table 3:** comparison of the sensitivity, specificity, and kappa values of laboratory staff performed microscopy and RDTs versus expert microscopy

Method	Sensitivity (%) (95%CI)	Specificity (95%CI)	Kappa
Clinical diagnosis	17.2 (8.59-29.43)	86.50 (83.88-88.88)	0.027
Microscopy	68.79 (55.46-80.46)	98.92 (97.88-99.53)	0.737
CareStart RDT	91.38 (81.02-97.14)	94.87 (93.03-96.35)	0.683
Wondfo RDT	94.83 (85.62-98.2)	94.60 (92.72-96.12)	0.691

RDT: rapid diagnostic tests

**Influence of malaria test results on treatment:** out of the total of 689 who were suspected and sent to the laboratory for malaria tests, 107 returned to clinicians with a positive result either by expert microscopy, laboratory staff performed microscopy, or any of the RDTs used. Thus 582 participants submitted negative test results to clinicians. All participants with positive results received appropriate antimalaria drugs, however, 85(14.6%) were given antimalaria drugs even with negative test results. A higher proportion of children under 5 years (20.0%) with negative results received antimalaria drugs compared to participants above 5 years old (13.2%) with no significant difference (X^2^-3.53, p-0.06). The study therefore found an adherence rate of 87.67% to malaria test results among clinicians. Whilst there was a 100% compliance to positive test results, compliance to negative test results was 85.4% as shown in Table 4.

**Table 4:** distribution of participants with negative malaria test results prescribed antimalaria drugs according to age groups

Age group	Prescribed antimalarial drug		
	Yes (%)	No (%)	Total
<5	24 (20.0)	96 (80)	120
>5	61 (13.2)	401 (86.8)	462
Total	85 (14.6)	497 (85.4)	582

## Discussion

The results of this study suggests a relatively low burden of malaria among the study population compared to the prevalence of 18% found in an earlier study conducted in the study area [1]. The difference in the prevalence between the two studies could be due to the study design and the study population. Whilst the earlier study involved only children with or without symptoms, this current study involved both children and adults who presented with symptoms suggestive of malaria. A retrospective study conducted in a Ghanaian hospital in the Greater Accra region found a higher prevalence of malaria of 12.3% [11]. The retrospective study did not make use of expert microscopist unlike the present study. Microscopy is commonly regarded as the gold standard for malaria confirmation in clinical settings. Studies have shown that performance of routine microscopy depends on the expertise of the microscopist [12,13]. In this study, microscopy performed by laboratory staff had a sensitivity of 68.79% compared to expert microscopy even though there was substantial agreement between the two tests. The lower predictive indices obtained by laboratory staff performed microscopy could be due to lack of expertise in preparing and reading of blood films, or a heavy work load that might affect the time a staff spent on a slide during microscopy. These possible contributory factors need to be investigated so as to improve the performance of routine microscopy performed by health staff. The laboratory staffs could also benefit from effective supportive supervision by experts. Previous studies have demonstrated that laboratory staff´s competency in parasite detection improved with supportive supervision [14].

The obviously subjective nature of microscopy is an indication that other parasitological tests must be employed to improve diagnosis of malaria in routine clinical settings. This study therefore evaluated the performance of two RDT kits as compared to expert microscopy. The high predictive indices exhibited by both RDT kits in this study is similar to what was found in a Kenyan study [10]. The RDTs were found to be more sensitive than microscopy performed by laboratory staff. The study findings are consistent with some previous studies [15,16], that also found that under field conditions RDTs perform better than microscopy. Since RDTs are simple to perform, quick and cost-effective they can be reliably be used to diagnose malaria as demonstrated by findings of this study. The HRP2-based RDT kits used in this study have been found in a systematic review [17] to have a sensitivity of 95.0% (95% CI: 93.5-96.2%) and a specificity of 95.2% (95% CI: 93.4-99.4). However, the *pf*HRP 2 detecting RDTs have the potential to overestimate malaria prevalence. This is because, the HRP 2 antigens can persist for up to 2 weeks after treatment [18]. Again the presence of the rheumatoid factor was shown to give false positive results in an earlier version of *p*fHRP 2 which has since been corrected [19]. There has been reports of false negatives by HRP 2 kits as a result of mutated or deleted genes [20,21].

Clinical diagnosis of malaria in this study had a sensitivity of 17.2% which is very low compared to that of laboratory staff performed microscopy and rapid diagnostic tests. This study findings suggests that over 80% of all managed empirically for malaria did not actually have malaria. Such low sensitivities for clinical diagnosis were also found in some earlier studies [22,23]. A study conducted among Gambian children that used a scoring system to assist peripheral health workers in making malaria diagnosis, developed a model that predicted clinical malaria with a sensitivity of 89% and a specificity of 61%, compared to those obtained by an experienced paediatrician without laboratory support [24]. The use of such restrictive criteria may increase the sensitivities of clinical diagnosis. However, WHO reports that a review of ten studies that had used more restrictive criteria in clinical algorithms found an increased probability of missing malaria infections [25]. In an earlier study among clinicians in this study area [5], many clinicians had reported a very high reliance on their clinical suspicion of malaria. Findings of this study clearly shows that such trust in clinical diagnosis could lead to poor management of patients and should therefore be discouraged. There was a high (87.7%) compliance to malaria test results in this study. In a meta-analysis investigating health workers´ compliance to malaria RDT test results, a similar rate of 83% was found [26]. Some other studies have reported lower compliance to malaria test results by clinicians [3,27]. The current study however found that compliance to negative test results was lower at 85.4% compared to a 100% adherence to positive test results. Many other studies have reported poor adherence to negative test results. In a Zambian study [28], 58.4% of patients with negative microscopy test results were prescribed anti-malaria drugs whilst in a study conducted in Ghana, 84.1% of patients with negative malaria test results obtained anti-malaria drugs [3]. Several studies have sought to find reasons for this practice among clinicians. Some of the reasons for this practice include mistrust of test results [29], lack of an alternative diagnosis [9], persistence of symptoms, patient pressure and demand [8].

**Limitations:** this study has some few limitations. The study did not make use of polymerase chain reaction which is known to have the highest sensitivity in the diagnosis of malaria to assess the diagnostic accuracy of the malaria RDT kits as well as the competence of the laboratory staff involved in the study. This was however, minimized by the use of highly trained experts in malaria diagnosis. Again, the study did not investigate what could possibly cause the non-malaria fevers among participants who tested negative to the parasitological tests.

## Conclusion

Findings from this study suggests that when microscopy and rapid diagnostic tests are performed by health workers in routine clinical settings, they compare favourably with expert microscopy. Clinical diagnosis on the other hand was found to be highly unreliable with poor predictive indices. Clinicians continue to prescribe antimalaria drugs for patients with negative malaria test results.

**Recommendations:** to improve the diagnostic accuracy of malaria in clinical settings and therefore increase clinicians trust in malaria test results, both microscopy and rapid diagnostic tests should be performed if possible, for all suspected cases of malaria. Health facilities should develop and implement quality assurance programs that will ensure that optimum quality parasitological tests for malaria are performed by health workers. There should be regular training on malaria tests and case management for clinicians as well as laboratory staff. Clinicians should especially be trained on management of non-malarial febrile conditions and the harmful effects of the over reliance on clinical findings only in the diagnosis of malaria.

### What is known about this topic

- Some clinicians do not trust malaria test results due to perceived incompetence of routine laboratory staff in the performance of the various malaria tests;
- Some clinicians diagnose and manage malaria without parasitological evidence.

### What this study adds

- Parasitological tests performed by routine laboratory staff are comparable to those performed by experts and could be reliably used to manage malaria;
- Clinical diagnosis of malaria cannot be relied upon to accurately diagnose malaria even in malaria endemic regions.
